# The Key to Phaco-Refractive Surgery: Does a Small Incision Make a Big Difference?

**DOI:** 10.7759/cureus.98053

**Published:** 2025-11-28

**Authors:** Shaibaan Mulla, Monika Kapur, Niharika Chaudhary, Sugourab Das, Vishnu Gupta, Hina Kauser, Taskin Khan, Mayuresh Naik

**Affiliations:** 1 Vitreo Retina Surgery, Aravind Eye Hospital, Chennai, IND; 2 Ophthalmology, Hamdard Institute of Medical Sciences and Research (HIMSR) and Hakeem Abdul Hameed Centenary (HAHC) Hospital, New Delhi, IND; 3 Ophthalmology, Bristol Eye Hospital, Bristol, GBR

**Keywords:** corneal astigmatism, opposite clear corneal incision, phacoemulsification cataract surgery, phaco-refractive surgery, surgically induced astigmatism

## Abstract

Purpose

To study and compare the efficacy of 2.8 mm single clear corneal incision versus 2.8 mm paired clear corneal incision on pre-existing astigmatism in patients undergoing phacoemulsification cataract surgery.

Methods

A prospective randomized double-blinded interventional comparative study was conducted at a tertiary healthcare center. The patients undergoing unilateral cataract surgery were divided into two groups: group A, patients undergoing phacoemulsification through a 2.8 mm single clear corneal incision (CCI), and group B, patients undergoing phacoemulsification through 2.8 mm opposite clear corneal incisions (OCCIs). Postoperative assessment was done at one day, one week, three weeks, and three months to note uncorrected visual acuity (UCVA), best-corrected visual acuity (BCVA), ophthalmic examination, corneal keratometry, and surgically induced astigmatism.

Results

A total of 130 eyes of 130 patients were included in our study. The change in corneal astigmatism was statistically significant (p = 0.04) in the paired OCCI group, while it was not statistically significant (p = 0.542) in the single CCI group at the end of three months. On comparing the change between the two groups, the paired OCCI group induced a greater degree of astigmatic change than the single CCI at three months, and it was statistically significant (p = 0.03).

Conclusion

There was a significant reduction in postoperative corneal astigmatism at three months with 2.8 mm paired OCCI, and the intergroup comparison was statistically significant. A 2.8 mm paired OCCI induces more surgical astigmatism at the end of three months than a 2.8 mm single clear corneal incision.

## Introduction

Modern-day cataract surgery aims at providing rapid visual rehabilitation and best uncorrected visual acuity (UCVA) with minimal postoperative astigmatism. It is estimated that about 63% of the population has preoperative astigmatism ranging between 0.25D and 1.25D, whereas the prevalence of >1.25D astigmatism is 18% [1]. Residual corneal astigmatism after cataract surgery may result in suboptimal UCVA and may result in the patient being dependent on spectacle use for clear distant vision.

Corneal astigmatism after cataract surgery can be managed conservatively with appropriate refractive correction or corrected surgically, but it would be more appropriate to combine both procedures in one, if possible [2]. Various methods have been used to correct pre-existing corneal astigmatism like toric intraocular lens (IOL) [3] arcuate keratotomy [4], limbal relaxing incision (LRI) [5], clear corneal incisions (CCIs) [6,7], opposite clear corneal incision (OCCI) on the steep meridian [8-11], and excimer-based laser procedures like laser-assisted in situ keratomileusis (LASIK) [12,13] and photorefractive keratectomy [14].

The introduction of clear corneal incision has almost revolutionized modern cataract surgeries. Simple manipulation of the corneal incision (location, size, and shape) does indeed help the surgeon to tailor the outcome after surgery [15]. The addition of a paired clear corneal incision has an additive effect and enhances the flattening of the corneal curvature [8].

Paired clear corneal incision is a relatively simple technique requiring no additional instruments or surgical setup. Location of the incision is of crucial importance, as OCCI leads to flattening of the corneal curvature by the formation of a scar at the site of surgical incision.

Only a few studies to date have explored the potential role of a 2.8 mm single clear corneal incision (SCCI) versus a 2.8 mm paired clear corneal incision as a practical measure to correct mild to moderate pre-existing astigmatism (PEA). Our study aimed to address this gap in the literature.

## Materials and methods

The study was approved by the Institutional Ethics Committee and the Institutional Review Board of Hamdard Institute of Medical Sciences and Research. Written and informed consent was taken from all patients. All procedures performed in the study involving human participants were in accordance with the 1964 Declaration of Helsinki and its later amendments.

This prospective randomized double-blinded interventional comparative study was conducted in the eye department of a tertiary healthcare hospital in North India over a period of one year, from November 2023 to October 2024. Patients aged 50 years and above, with age-related cataract and pre-existing corneal astigmatism (0.25 to 3D) undergoing phacoemulsification surgery were included in the study. Patients with complicated cataract, irregular astigmatism, zonular dehiscence, history of previous intraocular inflammation, history of glaucoma or retinopathy were excluded from our study. A total of 130 eyes from 130 patients were enrolled and randomly assigned into two groups of 65 eyes each using a 00 table of random numbers. Group A underwent phacoemulsification through a 2.8 mm SCCI placed at the steepest corneal meridian as determined by keratometry (K) readings; 0° and 180° axes were marked preoperatively on the slit lamp with the patient in the upright position, and the steep axis was marked intraoperatively on the operating table using a Mendez ring. The incision was created 0.5 mm anterior to the limbus, forming a 1.5 mm tunnel within the clear cornea. Group B underwent phacoemulsification through a 2.8 mm self-sealing clear corneal incision made at the steepest meridian, followed by the creation of an OCCI 180° from the primary incision after intraocular lens (IOL) implantation. Coaxial irrigation/aspiration was performed in all cases. The same foldable hydrophobic acrylic posterior chamber IOL was implanted in both groups. All the surgeries were performed under topical anesthesia under all aseptic precautions by a single surgeon in group A and by another single surgeon in group B. All the patients received routine topical steroids and antibiotic eyedrops postoperatively for four weeks. UCVA, best corrected visual acuity (BCVA), refraction, and keratometry were recorded for all patients preoperatively and postoperatively.

Statistical analysis

This involved comparison of preoperative and postoperative keratometry values in both groups at postoperative day 1, day 7, three weeks, and three months. Moreover, the change in keratometry values brought about by the two treatments was compared. Statistical evaluation was done by Student's t-test for comparing inter-group quantitative variables, and intra-group quantitative variables were compared by paired t-test. Statistical Package for Social Sciences (SPSS) version 21.0 (IBM Corp., Armonk, NY) was used for analyzing the data using an Excel spreadsheet (Microsoft® Corp., Redmond, WA).

## Results

A total of 130 eyes of 130 patients satisfying the inclusion criteria were randomly allocated to groups A (CCI) and B (OCCI), with 65 eyes in each group. Mean age in group A was 64.77 ± 11 years, and mean age in group B was 64.87 ± 12.26 years. Of the 65 patients in the CCI group, 31 were male, and 34 were female, while in the OCCI group, 30 patients were male and 35 were female. Both groups were epidemiologically, demographically, and clinically comparable with no statistically significant differences.

Mean corneal astigmatism

The intra-group comparison for mean corneal astigmatism at three months postoperatively in the CCI group (p = 0.532) was not statistically significant, whereas it was significant in the OCCI group (p = 0.04) when compared to the respective preoperative values (Table 1).

**Table 1:** Comparison of mean corneal astigmatism between the two groups p-value = 0.532 when preoperative mean astigmatism is compared to three months postoperative astigmatism using an independent t-test in the CCI group. p-value = 0.04 when preoperative mean astigmatism is compared to three months postoperative astigmatism using an independent t-test in the OCCI group. CCI, clear corneal incision; OCCI, opposite clear corneal incision

	Mean astigmatism (D) ± SD	
Follow-up period	CCI group (65 eyes)	OCCI group (65 eyes)
Preoperatively	2.1 ± 0.56D	2.51 ± 0.26D
Postoperatively day 1	1.9 ± 0.51D	1.50 ± 0.44D
Postoperatively week 1	1.8 ± 0.76D	1.13 ± 0.72D
Postoperatively month 1	1.6 ± 0.74D	1.1 ± 0.67D
Postoperatively month 3	1.5 ± 0.71D	0.90 ± 0.60D

Refraction

The change in mean refraction in both CCI (p = 0.08) and OCCI (p = 0.055) groups was not found to be statistically significant at three months postoperative mark (Table 2).

**Table 2:** Comparison of refraction between the two groups p-value = 0.08 when preoperative refraction is compared to three months postoperative refraction using an independent t-test in the CCI group. p-value = 0.055 when preoperative refraction is compared to three months postoperative refraction using an independent t-test in the OCCI group. CCI, clear corneal incision; OCCI, opposite clear corneal incision

	Refraction (D) ± SD	
Follow-up period	CCI group (65 eyes)	OCCI group (65 eyes)
Preoperatively	1.80 ± 0.66D	1.54 ± 0.52D
Postoperatively day 1	1.7 ± 0.66D	1.54 ± 0.52D
Postoperatively week 1	1.55 ± 0.59D	1.05 ± 0.56D
Postoperatively month 1	1.48 ± 0.53D	0.77 ± 0.53D
Postoperatively month 3	1.40 ± 0.51D	0.63 ± 0.51D

Postoperative cylindrical prescription

Analysis of postoperative refraction showed that seven patients of the CCI group and 18 patients of the OCCI group achieved good UCVA (Snellen 6/6). Some patients (16) of the CCI group required a cylindrical correction of 1.00 to 1.50D, whereas some patients of the OCCI group (18) did not require any cylindrical correction postoperatively (Table 3). 

**Table 3:** Postoperative cylindrical prescription after three months

Cylinder (D)	Number of eyes	
	CCI group	OCCI group
Plano	7	18
<0.50	9	12
>0.50 to 1.00	9	15
>1.00 to 1.50	16	8
>1.50 to 2.00	14	8
>2.00	10	4

Postoperative visual acuity

Comparison between the postoperative visual acuity showed that there was no statistical difference in the mean UCVA three months post-surgery between the two groups. However, there was a slightly greater number of eyes with UCVA of 6/6 in the OCCI group as compared to the CCI group (Table 4).

**Table 4:** Visual acuity three months after surgery

Visual acuity	Number of eyes			
	CCI group		OCCI group	
	UCVA	BCVA	UCVA	BCVA
6/6	38	41	42	51
6/9	20	13	8	8
6/12	3	3	6	3
6/18	2	2	5	2
6/24	2	1	4	1

Surgically induced astigmatism (SIA)

Calculation of surgically induced astigmatism was done using vector analysis with the Warren Hill calculator for SIA. The mean value of SIA in group A (p = 0.038) as well as in group B (p = 0.042) was significant at three monthly follow-ups (Table 5).

**Table 5:** Comparison of surgically induced astigmatism between the two groups p-value = 0.038 when preoperative mean corneal astigmatism is compared to three months postoperative SIA (calculated using Warren Hill calculator) using an independent t-test in the CCI group. p-value = 0.042 when preoperative mean corneal astigmatism is compared to three months postoperative SIA (calculated using Warren Hill calculator) using an independent t-test in the OCCI group. CCI, clear corneal incision; OCCI, opposite clear corneal incision

Follow-up period	CCI group (65 eyes)	OCCI group (65 eyes)
Preoperatively existing corneal astigmatism	2.1 ± 0.56D	2.51 ± 0.26D
Postoperative day 1 SIA	0.87 ± 0.51	1.28 ± 0.44
Postoperative week 1 SIA	0.75 ± 0.59	1.20 ± 0.73
Postoperative month 1 SIA	0.68 ± 0.43	1.15 ± 0.44
Postoperative month 3 SIA	0.64 ± 0.31	0.91 ± 0.76

## Discussion

The presence of PEA reduces visual acuity and affects the quality of vision after cataract surgery. Several approved surgical methods exist for the correction of pre-existing astigmatism. Toric IOL is the most preferred method for correcting moderate to high regular corneal astigmatism (> 1D). Toric IOL implantation is associated with higher procedural costs and a comparatively steeper learning curve, largely due to the need for precise preoperative assessment and meticulous intraoperative alignment of the lens. However, the postoperative IOL decentration (the most common complication in 10 to 15% of patients) could render the surgeons’ efforts futile sometimes [3,5,16,17].

Arcuate keratotomy requires the use of a diamond knife, corneal marker, pachymetry, and nomogram [4]. The incision size is between 6 mm and 7 mm in length, and these are performed on the steep axis [18]. Arcuate keratotomy adjacent to the limbus, i.e., limbus relaxing incision (LRI), heals rapidly, leaving minimum postoperative astigmatism, while a similar incision away from the limbus, i.e., corneal relaxing incision (CRI), has long-term postoperative complications such as light sensitivity, foreign body sensation, and halos [13].

A CCI along the steepest meridian induces a flattening effect on that meridian. An identical CCI opposite the first CCI (OCCI) contributes to enhancing the corneal flattening and reducing the postoperative astigmatism after cataract surgery [8-11].

Previous studies of OCCIs have reported a mean reduction in corneal astigmatism ranging from 0.50 to 2.06D. Khokhar et al. concluded from their study that the mean astigmatic correction was 1.66 ± 0.50D in the group receiving 3.2 mm paired OCCI and 0.85 ± 0.75D in the group receiving 3.2 mm SCCI [18]. Ren et al. performed a randomized, controlled, prospective study in 140 patients to compare the effects of 2.0 mm and 3.0 mm SCCI and Paired OCCI on corneal astigmatism in 2019. The reduction in corneal astigmatism was 0.61 ± 0.38D in the 3.0 mm OCCIs group, which was significantly higher (p ≤ 0.004) than 0.29 ± 0.29D in the 2.0 mm OCCIs group. The mean SIA was 1.07 ± 0.51D in the 3.0 mm OCCIs group, higher than 0.61 ± 0.35D in the 2.0 mm OCCIs group (p = 0.001) [19]. A similar study done by Sethi et al. in 2017 inferred that reducing the incision size from 2.8 to 2.2 mm did not lead to any significant decrease in the SIA [20].

Our study concurs with these observations. We saw a mean corneal astigmatic change of 1.60D in patients with OCCIs and 0.6D in patients with SCCIs. The change in preoperative and postoperative corneal astigmatism value was not significant in the SCCI group (p = 0.542), whereas significant statistical differences were found at the end of three months in the paired OCCI group (p = 0.04). On comparing the change in keratometric readings between the SCCI group and the paired OCCI group, the change was statistically significant as well (p = 0.03). This showed that paired OCCI induced a higher degree of astigmatic change at the end of three months as compared to the SCCI group.

Previous studies using the OCCI technique observed an overall SIA between 1.4 and 2.25D after a postoperative period of two to three months [8-11,16,18]

Differences in astigmatic correction outcomes among surgeons using on-axis techniques are often due to the difficulty of aligning the incision precisely with the steep corneal meridian. Even small deviations in axis alignment can significantly reduce the procedure’s corrective effect, given the axis-sensitive nature of corneal refractive changes. In our study, SCCI surgeries were performed by a single surgeon, and opposite CCI surgeries were performed by another single surgeon. SIA of both surgeons was the same as calculated from previous data, and that nullifies the effect of both surgeons since the steps of surgery were defined prior to the study.

There was no significant difference in the incidence of intraoperative or postoperative complications between the two groups. The most frequently observed complications included posterior capsule rent (five eyes), which was managed with anterior vitrectomy followed by IOL implantation in the bag. 

CCIs have inherent drawbacks, such as increased risk of endophthalmitis and early wound-related complications. However, it still remains a popular technique due to procedural simplicity, requiring neither advanced surgical expertise nor specialized instrumentation. Their favorable predictability and contribution to corneal biomechanical stability make them a practical option for correcting mild to moderate PEA. Additionally, the use of OCCIs during phacoemulsification has been shown to enhance astigmatic correction beyond that achievable with SCCI approaches.

A key limitation of this study is the absence of a control group with comparable preoperative astigmatism and the relatively short follow-up period, which constrain the evaluation of the long-term efficacy of OCCIs for correcting preexisting astigmatism. However, the inclusion of a suitably matched control group poses methodological challenges, as randomization may not ensure equivalence in baseline astigmatic profiles, thereby limiting the ability to eliminate confounding variables.

## Conclusions

OCCI could be a safe, cost-effective, and predictable alternative to treat mild-to-moderate corneal astigmatism surgically, especially in developing countries with limited resources, financial constraints, and logistical difficulties of toric IOL availability and expensive laser setups. However, long-term follow-up might be warranted to ascertain the impact of OCCI on corneal integrity and regression of corneal curvature.
